# A real-world study was conducted to develop a nomogram that predicts the occurrence of anastomotic leakage in patients with esophageal cancer following esophagectomy

**DOI:** 10.18632/aging.205780

**Published:** 2024-05-01

**Authors:** Chenglin Li, Wei Song, Jialing Zhang, Zhongneng Xu, Yonggang Luo

**Affiliations:** 1 Department of Cardiothoracic Surgery, The Affiliated Huaian No. 1 People’s Hospital of Nanjing Medical University, Huaian, Jiangsu 223300, China; 2 Department of Gastroenterology, The Affiliated Huaian No. 1 People’s Hospital of Nanjing Medical University, Huaian, Jiangsu 223300, China

**Keywords:** inflammatory biomarkers, immunology, esophageal cancer, anastomotic leakage, target

## Abstract

Background: The incidence of anastomotic leakage (AL) following esophagectomy is regarded as a noteworthy complication. There is a need for biomarkers to facilitate early diagnosis of AL in high-risk esophageal cancer (EC) patients, thereby minimizing its morbidity and mortality. We assessed the predictive abilities of inflammatory biomarkers for AL in patients after esophagectomy.

Methods: In order to ascertain the predictive efficacy of biomarkers for AL, Receiver Operating Characteristic (ROC) curves were generated. Furthermore, univariate, LASSO, and multivariate logistic regression analyses were conducted to discern the risk factors associated with AL. Based on these identified risk factors, a diagnostic nomogram model was formulated and subsequently assessed for its predictive performance.

Results: Among the 438 patients diagnosed with EC, a total of 25 patients encountered AL. Notably, elevated levels of interleukin-6 (IL-6), IL-10, C-reactive protein (CRP), and procalcitonin (PCT) were observed in the AL group as compared to the non-AL group, demonstrating statistical significance. Particularly, IL-6 exhibited the highest predictive capacity for early postoperative AL, exhibiting a sensitivity of 92.00% and specificity of 61.02% at a cut-off value of 132.13 pg/ml. Univariate, LASSO, and multivariate logistic regression analyses revealed that fasting blood glucose ≥7.0mmol/L and heightened levels of IL-10, IL-6, CRP, and PCT were associated with an augmented risk of AL. Consequently, a nomogram model was formulated based on the results of multivariate logistic analyses. The diagnostic nomogram model displayed a robust discriminatory ability in predicting AL, as indicated by a C-Index value of 0.940. Moreover, the decision curve analysis provided further evidence supporting the clinical utility of this diagnostic nomogram model.

Conclusions: This predictive instrument can serve as a valuable resource for clinicians, empowering them to make informed clinical judgments aimed at averting the onset of AL.

## INTRODUCTION

Esophageal cancer (EC) represents a widespread malignant neoplasm on a global scale, with China bearing a particularly high burden of mortality and morbidity associated with this disease [1, 2]. The 5-year overall survival rate for individuals undergoing curative treatment for EC varies between 40% and 50% [3, 4]. The management of EC typically encompasses a multimodal approach, incorporating various strategies such as surgical resection, chemotherapy, radiotherapy, immunotherapy, and targeted therapy [2, 5–7]. Esophagectomy is widely recognized as the foremost therapeutic modality for EC, given its efficacy. Nevertheless, it is an invasive intervention that entails the potential for postoperative complications [8]. Anastomotic leakage (AL) represents a grave and potentially life-threatening complication that may arise following esophagectomy. Its occurrence is linked to heightened mortality and morbidity rates, prolonged hospital stays, and elevated financial burdens on both patients and healthcare systems [9–12]. In addition, the recurrence of EC is affected by AL [13]. The incidence of AL among patients with EC can vary, ranging from 11.4% to 21.2% [14–17]. The precise etiology of AL remains elusive. Nevertheless, various risk factors have been identified, including cardiac comorbidity, male gender, advanced age, diabetes mellitus, renal disease, higher American Society of Anesthesiologists (ASA) score, pulmonary comorbidity, vascular comorbidity, higher body mass index (BMI), and hypertension. These factors have been correlated with an elevated susceptibility to developing AL subsequent to esophagectomy [18–21]. Consequently, there exists a necessity to identify biomarkers that can facilitate the timely detection of AL in individuals with high-risk EC [22]. However, the clinical manifestations of AL often manifest at a later stage or display nonspecific features [11]; thus, it is difficult to predict AL in EC patients at an early stage.

The timely prediction of AL has the potential to considerably enhance the quality of life for individuals diagnosed with EC. Moreover, it can exert a positive influence on survival rates and recurrence, thereby contributing to improved patient outcomes [23]. A plethora of biomarkers have been extensively investigated to prognosticate the incidence of AL subsequent to esophagectomy [24–30], including C-reactive protein (CRP) or procalcitonin (PCT) [31–35]. Plasma cytokine levels have shown promise as potential predictors of AL [36–39] and reportedly have greater diagnostic abilities for AL, compared with CRP or PCT [37].

There exists a critical need for reliable preoperative predictive models to identify patients at risk of anastomotic leakage, enabling early intervention and personalized treatment strategies. Addressing this gap in the literature, this study aims to develop and validate a novel predictive model for anastomotic leakage in esophageal cancer patients, with the ultimate goal of improving clinical outcomes and enhancing patient care.

## MATERIALS AND METHODS

### Patients

For this particular investigation, a total of 438 patients diagnosed with EC were enrolled, and they were admitted to the Affiliated Huaian No. 1 People’s Hospital of Nanjing Medical University during the period spanning September 2020 to March 2022. Inclusion criteria encompassed an age exceeding 18 years, confirmed diagnosis of EC supported by pathological evidence, and having undergone esophagectomy. Exclusion criteria entailed the presence of concurrent malignancies, infectious diseases, autoimmune disorders, or inadequate availability of data. The diagnosis of AL was established through the utilization of various criteria, including the identification of contrast extravasation on postoperative computed tomography, leakage of gastrointestinal tract contents via a wound or drainage tube, appearance of blue liquid in the incision or pleural drainage following oral administration of methylene blue, and endoscopic observations indicative of AL. The diagnosis of AL among EC patients occurred within the time frame ranging from the 3^rd^ to the 10^th^ day postoperatively. The severity of AL was classified into three grades: grade A, necessitating no active therapeutic intervention; grade B, requiring active therapeutic interventions excluding surgery; and grade C, necessitating surgical intervention. Informed consent was obtained from all participants, and the study protocol obtained approval from the Ethics Committee of Huaian No. 1 People’s Hospital, adhering to the principles outlined in the Helsinki Declaration.

### Measurement of cytokine levels

Cytometric bead array analysis was conducted using the Human Th1/Th2 CBA kit from JIANGXI CELLCENE BIOTECH Co., Ltd., following the manufacturer’s instructions. Flow cytometry was employed to quantify the cytokine levels in individuals diagnosed with EC following esophagectomy. All cytokine levels were assessed on the first day postoperatively.

### Statistical analysis

The collected data were subjected to various statistical analyses. Continuous variables were evaluated using Student’s *t*-test, while the chi-squared test was employed for categorical variables. The Mann-Whitney *U*-test was applied for nonparametric variables. Receiver Operating Characteristic (ROC) curves were constructed to assess the diagnostic capabilities of the biomarkers for AL. Univariate, LASSO, and multivariate logistic regression analyses were performed to identify the risk factors associated with AL. Variables that yielded a *p*-value of less than 0.05 in the univariate analyses were included in the multivariate regression analysis. Odds ratios (OR) and their corresponding 95% confidence intervals (CI) were computed. A significance level of *p* < 0.05 was considered statistically significant. The statistical analyses were conducted using GraphPad Prism (version 8.0, La Jolla, CA, USA), SPSS (version 21.0, Chicago, IL, USA), R software (version 4.1.3), and MedCalc software.

## RESULTS

### Clinicopathological variables in EC patients

**Figure 1:**
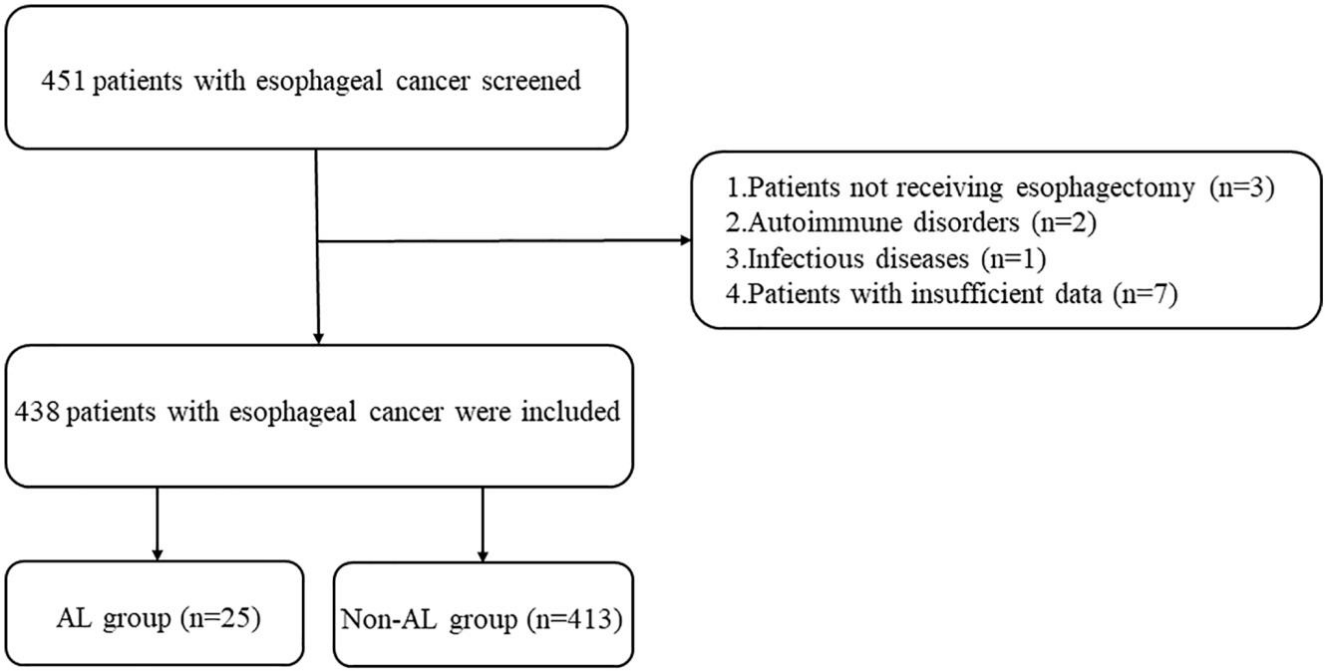
Study flowchart.

**Figure 2:**
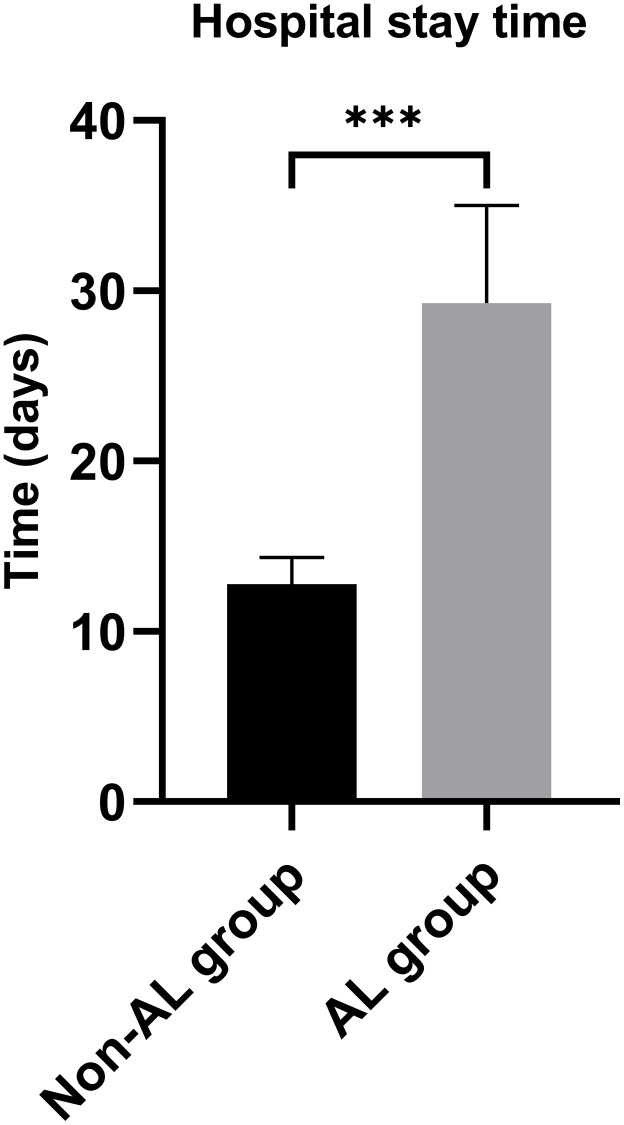
**Hospital stay time between anastomotic leakage group and non-anastomotic leakage group.** ^***^P < 0.001.

The study enrolled a total of 438 patients diagnosed with EC, as illustrated in Figure 1. Among them, 25 patients experienced AL. The clinicopathological characteristics of the EC patients are presented in Table 1. Notably, the AL group displayed a considerably prolonged hospitalization duration in comparison to the non-AL group, as depicted in Figure 2 (29.28 ± 5.73 days vs. 12.78 ± 1.58 days, *p* < 0.001). Age (62.00 ± 9.77 years vs. 57.56 ± 9.84 years), smoking status, and the presence of fasting blood glucose (FBG) exhibited significant disparities between the two groups (Table 2). No other variables demonstrated significant differences between the AL and non-AL groups. The severity of AL was categorized into grades A, B, and C, encompassing 3, 21, and 1 patient(s) in each respective category (Supplementary Table 1); the variations in the levels of CRP, PCT, IL-6, and IL-10 among these three groups are shown in Supplementary Table 2.

**Table 1:** The baseline characteristics of esophageal cancer patients.

**Variables**	**Esophageal cancer (*n* = 438)**
Age (years)	57.82 ± 9.88
Sex	
Male	288
Female	150
Smoking	
Yes	285
No	153
Drinking	
Yes	215
No	223
BMI (kg/m^2^)	22.49 ± 3.10
Hypertension	
Yes	100
No	338
Fasting blood glucose	
≥7.0 mmol/L	67
<7.0 mmol/L	371
Family history	
Yes	123
No	315
Pathological grading	
Well differentiation	97
Moderate differentiation	180
Poorly differentiation	161
Histological type	
Squamous cell carcinoma	419
Undifferentiated carcinoma	9
Others	10
TNM stage	
I	100
II	257
III	81
Tumor location	
Upper	53
Middle	240
Lower	96
Gastroesophageal junction	49
Level of anastomosis	
Intra thoracic	69
Neck	369
Surgical type	
Open surgery	17
Thoracoscopy	421
Hospital stay time (days)	13.73 ± 4.34

Abbreviations: BMI: body mass index; TNM: tumor node metastasis.

**Table 2:** The baseline characteristics of esophageal cancer patients between AL and non-AL groups.

**Variables**	**AL group (*n* = 25)**	**Non-AL group (*n* = 413)**	***P***
Age (years)	62.00 ± 9.77	57.56 ± 9.84	**0.041**
Sex			0.532
Male	15	273	
Female	10	140	
Smoking			0.013
Yes	22	263	
No	3	150	
Drinking			0.911
Yes	12	203	
No	13	210	
BMI (kg/m^2^)	21.80 ± 2.72	22.53 ± 3.12	0.253
Hypertension			0.261
Yes	8	92	
No	17	321	
Fasting blood glucose			**0.001**
≥7.0 mmol/L	10	57	
<7.0 mmol/L	15	356	
Family history			0.992
Yes	7	116	
No	18	297	
Pathological grading			0.465
Well differentiation	4	93	
Moderate differentiation	9	171	
Poorly differentiation	12	149	
Histological type			0.108
Squamous cell carcinoma	22	397	
Undifferentiated carcinoma	1	8	
Others	2	8	
TNM stage			0.599
I	4	96	
II	17	240	
III	4	77	
Tumor location			0.889
Upper	3	50	
Middle	13	227	
Lower	5	91	
Gastroesophageal junction	4	45	
Level of anastomosis			**0.044**
Intra thoracic	8	61	
Neck	7	352	
Surgical type			**0.012**
Thoracotomy	4	13	
Thoracoscopy	21	400	
Hospital stay time (days)	29.28 ± 5.73	12.78 ± 1.58	**<0.001**

Abbreviations: BMI: body mass index; TNM: tumor node metastasis; AL: anastomotic leakage. Bold values are statistically significant (*P* < 0.05).

### Predictive powers of inflammatory biomarkers for AL

**Figure 3:**
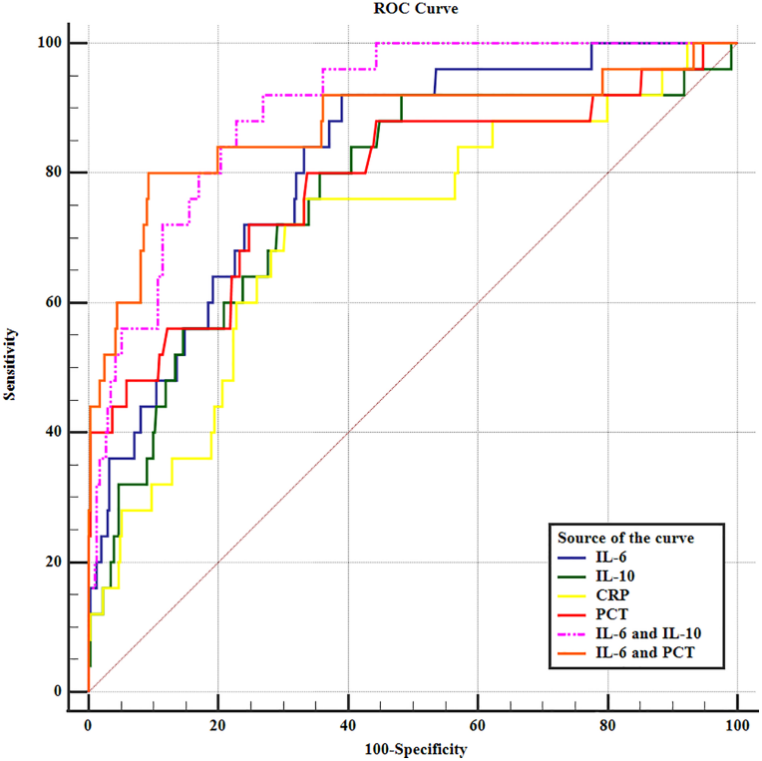
The receiver operating characteristic curve of inflammatory biomarkers in predicting anastomotic leakage in EC patients.

On the first day postoperatively, the levels of interleukin-6 (IL-6), IL-10, C-reactive protein (CRP), and procalcitonin (PCT) were assessed in both the AL and non-AL groups, as outlined in Table 3. Remarkably elevated levels of IL-6 (203.01 ± 61.20 vs. 129.20 ± 54.66 pg/ml), IL-10 (15.08 ± 7.77 vs. 8.76 ± 5.02 pg/ml), CRP (186.11 ± 60.60 vs. 144.23 ± 45.27 mg/l), and PCT (4.84 ± 2.98 vs. 2.50 ± 1.03 ng/ml) were observed in the AL group compared to the non-AL group (Table 3). The ROC curve analysis presented in Table 4 indicated that IL-6 exhibited the highest predictive value as an early postoperative indicator for AL, demonstrating a sensitivity of 92.00% and specificity of 61.02% at a cutoff value of 132.13 pg/ml. These findings suggest that IL-6 possesses favorable diagnostic ability for AL. Furthermore, the combination of IL-6 and IL-10 exhibited superior diagnostic capability for AL compared to either cytokine alone or other combinations of inflammatory biomarkers (Figure 3). The area under the curve (AUC) for the combination of IL-6 and IL-10 was 0.899 (95% confidence interval = 0.87–0.93, *p* < 0.001).

**Table 3:** Cytokine levels in the AL and non-AL groups on postoperative day 1.

**Variables**	**AL (*n* = 25)**	**Non-AL (*n* = 413)**	***P***
IL-6 (pg/ml)	203.01 ± 61.20	129.20 ± 54.66	**<0.001**
IL-10 (pg/ml)	15.08 ± 7.77	8.76 ± 5.02	**<0.001**
CRP (mg/l)	186.11 ± 60.60	144.23 ± 45.27	**<0.001**
PCT (ng/ml)	4.84 ± 2.98	2.50 ± 1.03	**0.001**

Abbreviations: IL-6: interleukin-6; IL-10: interleukin-10; CRP: C-reactive protein, PCT: Procalcitonin; AL: anastomotic leakage. Bold values are statistically significant (*P* < 0.05).

**Table 4:** ROC curves for predictive values of serum inflammatory cytokines in detecting anastomotic leakage in patients with esophageal cancer.

**Variables**	**Youden index J**	**SE**	**Associated criterion**	**Sensitivity %**	**Specificity %**	**AUC (95%)**	**95% CI**	**Significance level *P* (Area = 0.5)**
IL-6 (pg/ml)	0.530	0.040	>132.13	92.00	61.02	0.818	0.78–0.85	<0.001
IL-10 (pg/ml)	0.444	0.053	>9.97	80.00	64.41	0.767	0.73–0.81	<0.001
CRP (mg/l)	0.428	0.056	>162.97	76.00	66.83	0.712	0.67–0.75	<0.001
PCT (ng/ml)	0.473	0.056	>3.12	72.00	75.30	0.786	0.74–0.82	<0.001
CRP and PCT	0.564	0.049	>0.056	76.00	80.39	0.840	0.80–0.87	<0.001
IL-10 and CRP	0.549	0.042	>0.060	76.00	78.93	0.832	0.79–0.87	<0.001
IL-10 and PCT	0.608	0.043	>0.068	76.00	84.75	0.861	0.83–0.89	<0.001
IL-6 and CRP	0.615	0.043	>0.057	84.00	77.48	0.844	0.81–0.88	<0.001
IL-6 and IL-10	0.652	0.025	>0.043	88.00	77.24	0.899	0.87–0.93	<0.001
IL-6 and PCT	0.708	0.048	>0.088	80.00	90.80	0.872	0.84–0.90	<0.001

Abbreviations: ROC: Receiver Operating Characteristic; SE: stand error; AUC: Area Under the ROC Curve; 95% CI: Confidence interval; IL-6: interleukin-6; IL-10: interleukin-10; CRP: C-reactive protein, PCT: Procalcitonin.

### Risk factors for AL

**Figure 4:**
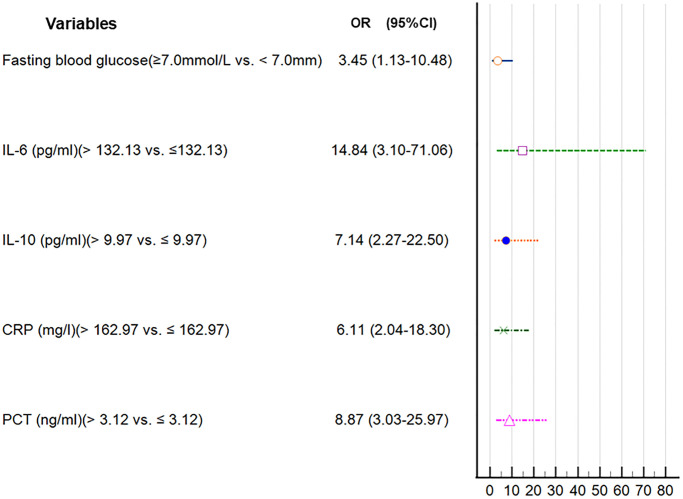
Forest plot of the parameters in the multivariate regression analysis.

The univariate logistic analysis revealed several factors that were associated with the risk of AL. These factors included older age (>60 vs. ≤60 years), smoking (yes vs. no), FBG (≥7.0 mmol/L vs. <7.0 mmol/L), intrathoracic location (intrathoracic vs. neck), open surgery (open surgery vs. thoracoscopy), and higher levels of IL-10, IL-6, CRP, and PCT (Table 5). Next, we identified five candidate predictors by LASSO regression analysis: FBG, IL-6, IL-10, CRP, and PCT (Supplementary Figure 1). In the multivariate analysis, after adjusting for other variables, it was found that FBG ≥7.0 mmol/L, IL-6 >132.13 pg/ml, IL-10 >9.97 pg/ml, CRP >162.97 mg/l, and PCT >3.12 ng/ml were independent risk factors for AL (Table 6 and Figure 4).

**Table 5:** Risk factors for anastomotic leakage in patients with esophageal cancer by univariate logistic regression analyses.

**Variables**	**B**	**S.E.**	**Wald**	**OR (95% CI)**	***P***
Age (years)					
>60 vs. ≤60	0.884	0.421	4.418	2.42 (1.06–5.52)	**0.036**
Sex					
Male vs. Female	−0.262	0.421	0.388	0.77 (0.34–1.76)	0.533
Smoking					
Yes vs. No	1.431	0.624	5.260	4.18 (1.23–14.21)	**0.022**
Drinking					
Yes vs. No	−0.046	0.412	0.013	0.96 (0.43–2.14)	0.911
BMI					
>24 vs. ≤24	−0.824	0.556	2.194	0.44 (0.15–1.31)	0.139
Hypertension					
Yes vs. No	0.496	0.445	1.243	1.64 (0.69–3.93)	0.265
Fasting blood glucose					
≥7.0 mmol/L vs. <7.0 mmol/L	1.426	0.432	10.879	4.16 (1.78–9.72)	**0.001**
Family history					
Yes vs. No	−0.004	0.459	<0.001	0.99 (0.41–2.45)	0.992
Pathological grading					
Moderate vs. Well	0.202	0.625	0.108	1.22 (0.37–4.08)	0.743
Poorly vs. Well	0.627	0.592	0.122	1.87 (0.59–5.98)	0.290
Histological type					
Undifferentiated carcinoma vs. Squamous cell carcinoma	0.813	1.083	0.564	2.26 (0.27–18.84)	0.453
Others vs. Squamous cell carcinoma	1.507	0.820	3.373	4.51 (0.90–22.52)	0.066
TNM stage					
II vs. I	0.531	0.569	0.871	1.70 (0.56–5.18)	0.351
III vs. I	0.221	0.723	0.093	1.25 (0.30–5.15)	0.760
Tumor location					
Lower vs. Upper	−0.047	0.659	0.005	0.95 (0.26–3.48)	0.944
Middle vs. Upper	−0.088	0.751	0.014	0.92 (0.21–3.99)	0.907
Gastroesophageal junction vs. Upper	0.393	0.791	0.247	1.48 (0.31–6.98)	0.619
Location of anastomotic leakage					
Intra thoracic vs. Neck	0.999	0.451	4.915	2.72 (1.12–6.57)	0.027
Surgical type					
Open surgery vs. Thoracoscopy	1.768	0.614	8.293	5.86 (1.76–19.53)	0.004
IL-6 (pg/ml)					
>132.13 vs. ≤132.13	2.890	0.744	15.089	18.00 (4.19–77.38)	<0.001
IL-10 (pg/ml)					
>9.97 vs. ≤9.97	1.979	0.510	15.036	7.24 (2.66–19.68)	**<0.001**
CRP (mg/l)					
>162.97 vs. ≤162.97	1.853	0.480	14.916	6.38 (2.49–16.34)	**<0.001**
PCT (ng/ml)					
>3.12 vs. ≤3.12	2.059	0.460	20.057	7.84 (3.18–19.31)	**<0.001**

Abbreviations: IL-6: interleukin-6; IL-10: interleukin-10; CRP: C-reactive protein, PCT: Procalcitonin; BMI: body mass index; TNM: tumor node metastasis. Bold values are statistically significant (*P* < 0.05).

**Table 6:** Risk factors for anastomotic leakage in patients with esophageal cancer by multivariate logistic regression analyses.

**Variables**	**B**	**S.E.**	**Wald**	**OR (95% CI)**	***P***
Fasting blood glucose					
≥7.0 mmol/L vs. <7.0 mmol/L	1.237	0.567	4.760	3.45 (1.13-10.48)	**0.029**
IL-6 (pg/ml)					
>132.13 vs. ≤132.13	2.698	0.799	11.398	14.84 (3.10-71.06)	**0.001**
IL-10 (pg/ml)					
>9.97 vs. ≤9.97	1.966	0.585	11.276	7.14 (2.27-22.50)	**0.001**
CRP (mg/l)					
>162.97 vs. ≤162.97	1.809	0.560	10.438	6.11 (2.04-18.30)	**0.001**
PCT (ng/ml)					
>3.12 vs. ≤3.12	2.182	0.548	15.839	8.87 (3.03-25.97)	**0.000**

Abbreviations: IL-6: interleukin-6; IL-10: interleukin-10; CRP: C-reactive protein, PCT: Procalcitonin. Bold values are statistically significant (*P* < 0.05).

### Nomogram model of tredicting the AL

**Figure 5:**
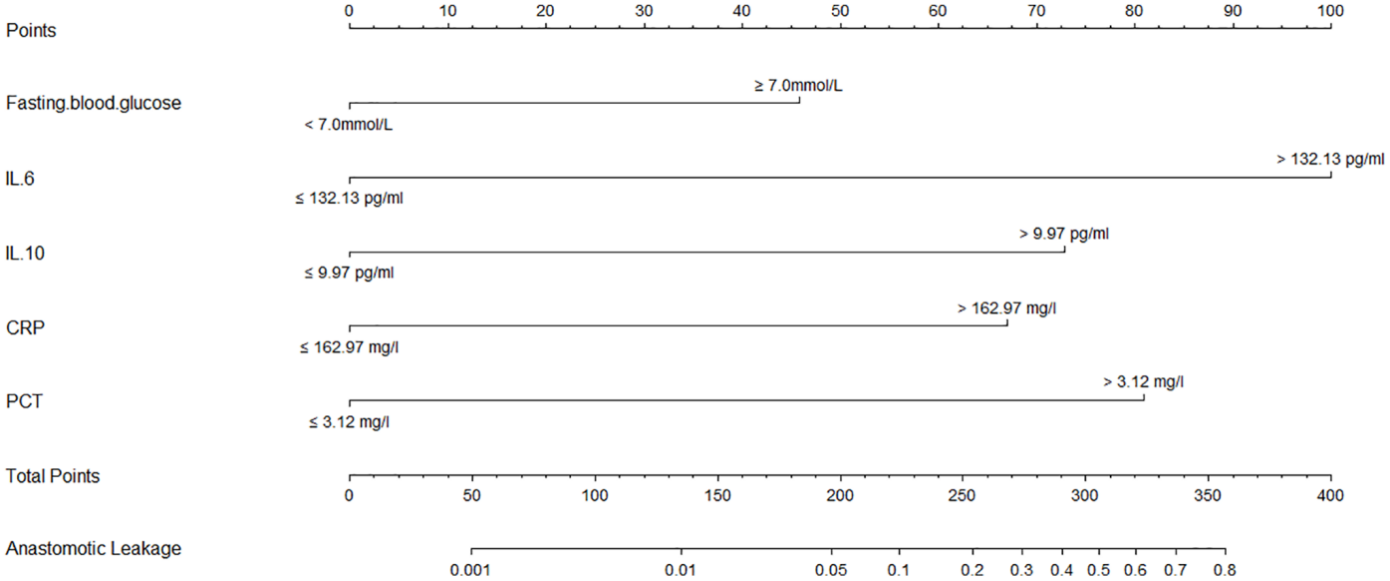
Calibration and clinical use of a diagnostic nomogram for predicting anastomotic leakage in patients with EC.

**Figure 6:**
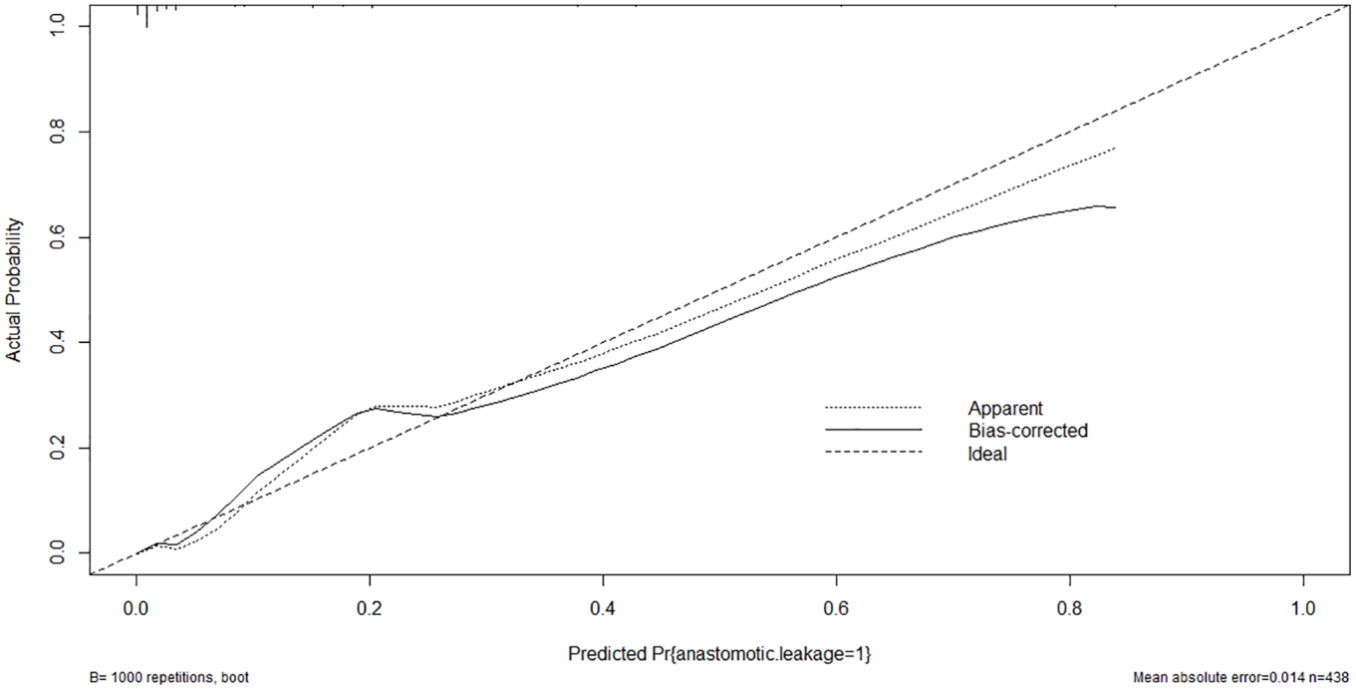
Calibration curve of the nomogram model of anastomotic leakage in patients with EC.

**Figure 7:**
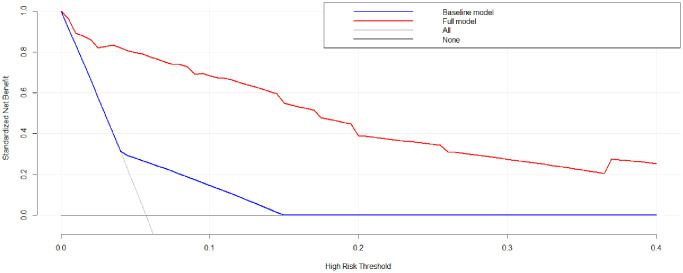
Decision curve analysis of the nomogram model of anastomotic leakage in patients with EC.

A nomogram model was developed based on the results of the multivariate logistic regression analysis to predict the occurrence of AL in patients with EC, as depicted in Figure 5. The C-Index of this predicting nomogram was calculated to be 0.940 (95% CI = 0.901–0.978), indicating a good discriminative ability of the model (Figure 6). Additionally, the decision curve analysis (DCA) demonstrated the clinical utility of this diagnostic nomogram model for making informed clinical decisions (Figure 7).

## DISCUSSION

This study revealed that the levels of IL-10, IL-6, CRP, and PCT in the peripheral blood were significantly elevated in the group of patients who experienced AL following esophagectomy, compared to the group without AL. Among these biomarkers, IL-6 exhibited the highest predictive value for AL, as indicated by the area under the receiver operating characteristic (ROC) curve. Moreover, combining IL-10 and IL-6 resulted in improved predictive power for AL, surpassing the individual cytokines or other combinations of inflammatory biomarkers. Logistic regression analyses demonstrated that FBG ≥7.0 mmol/L and higher levels of IL-10, IL-6, CRP, and PCT were associated with an increased risk of AL. Furthermore, the developed diagnostic nomogram model, based on these identified risk factors, proved to be effective in predicting the occurrence of AL in patients undergoing esophagectomy.

The diagnosis of AL is primarily based on the observation of clinical symptoms and the interpretation of imaging studies. Clinical symptoms may include fever, increased heart rate, chest or abdominal pain, difficulty swallowing, and signs of infection such as wound redness or drainage. Imaging studies, such as computed tomography (CT) scans or contrast studies, are commonly used to visualize the anastomotic site and detect any signs of leakage, such as contrast extravasation or the presence of fluid in areas outside the surgical site. These diagnostic modalities play a crucial role in confirming the presence of AL and guiding subsequent management decisions [11]. The pathophysiology of the disease remains unclear, with the development of AL after esophagectomy being associated with factors such as adequate perioperative preparation, as well as technical and anesthesiological considerations [40, 41]. The diagnosis of AL is often delayed due to the nonspecific and heterogeneous nature of its clinical manifestations [41]. A technical defect may be responsible for the occurrence of early AL, while late AL could be attributed to occult clinical symptoms in the early stages or an increase in oral intake after discharge [26, 42]. The timely diagnosis of AL is crucial, emphasizing the need for new biomarkers to aid in its detection.

CRP and PCT are biomarkers of AL [38, 43–45]. Elevated levels of C-reactive protein (CRP), an acute-phase protein, can be observed in both infectious and non-infectious conditions. However, due to its elevation in both situations, the CRP level lacks effectiveness in effectively distinguishing between infection and surgical complications [46, 47]. PCT, when compared to CRP, demonstrates greater specificity as a marker for severe infections and complications [48–50]. The ability of PCT to differentiate among postoperative complications is currently unknown and requires further investigation [49, 51]. According to the study by Lagoutte et al., the accuracy of PCT in predicting AL was found to be lower compared to CRP [49].

According to the findings of Ellebaek et al., postoperative cytokine levels, specifically IL-10 and IL-6, were observed to increase in patients who developed AL within 5 days after surgery. These elevated levels were found to be predictive of AL [52]. In the study conducted by Zawadzki et al., it was suggested that IL-6 served as a superior predictor of AL compared to CRP, particularly after low anterior resection for rectal cancer [37]. Certain studies have proposed that an increased peritoneal level of IL-6 exhibits stronger predictive capabilities for the occurrence of AL following colorectal surgery [36, 39, 53–56]; however, it is important to note that these studies did not measure the level of IL-6 in peripheral blood. In a study by Song et al., it was reported that plasma levels of IL-10, IL-6, and IL-8 on postoperative day 1 can serve as predictive markers for the development of AL in patients with EC undergoing esophagectomy [24]. Furthermore, IL-10 demonstrated higher predictive accuracy for AL compared to IL-6 or IL-8, as reported by Song et al. [24]. In the present study, peripheral levels of IL-10, IL-6, CRP, and PCT were significantly higher in the AL group than in the non-AL group after esophagectomy. Based on the area under the ROC curve, IL-6 was the best predictor of AL, which was inconsistent with a previous report [24]. This discrepancy may be related to the use of different definitions of AL and/or different cytokine assay methods; it may also be related to heterogeneity in the study populations.

In addition, the combination of IL-10 and IL-6 showed greater predictive power for AL, compared with either cytokine alone or other combinations of cytokines (Table 3). The mechanism underlying the association among IL-6, IL-10, and AL is unclear. The levels of IL-10 and IL-6 are elevated in sepsis [57–59], indicating a mixed hyperinflammatory and immunosuppressed status. AL after esophagectomy is associated with severe infection; thus, it is similar to sepsis. IL-10 promotes survival in mice with septic peritonitis [60], suggesting an anti-inflammatory effect. Peritoneal levels of IL-10 and IL-6 are reportedly predictive of AL after colorectal surgery [55], which was consistent with our findings. Cytokines are primarily divided into anti-inflammatory and proinflammatory cytokines. IL-6 and IL-10 were proinflammatory and anti-inflammatory cytokines, respectively. Greca et al. suggested that IL-6 showed a detrimental influence on the healing of colonic anastomoses [61] and may trigger AL. The serum level of CRP is a good indicator of symptomatic AL [62]; it is elevated in response to an increased level of IL-6. In addition, an elevated peritoneal level of IL-6 is associated with an increased risk of AL [36, 55, 56]. IL-10 induces an immunosuppressive or anti-inflammatory response and maintains inflammatory homeostasis in AL [31, 56]. IL-10 could facilitate innate immune responses to reduce the damage caused by bacterial and viral infections [63]. It also promotes tissue healing after inflammation or infection [64, 65]. IL-10 thus reflects the anti-inflammatory response to postoperative infection and surgical injury, which contributes to postoperative complications such as AL [66].

In addition, patient-related factors including diabetes mellitus, old age, alcohol, obesity, male sex, steroid use, smoking, malnutrition, and radiation therapy are reportedly associated with an elevated risk of AL [67–71]. In this study, univariate, LASSO, and multivariate logistic analyses indicated that FBG ≥7.0 mmol/L, and higher levels of IL-10, IL-6, CRP, and PCT were risk factors for AL. According to the results of logistic regression analysis, a diagnostic nomogram model was developed. This diagnostic nomogram model had a good discriminative ability for AL with a C-index value of 0.940. In addition, DCA showed that this diagnostic nomogram model was useful for making clinical decisions.

This study had several limitations. First, we evaluated the predictive powers of IL-6 and IL-10 for AL in EC patients; other cytokines should be investigated, especially for IL-8. Second, this study had a retrospective design. Third, subclinical AL was not routinely evaluated. Fourth, we did not investigate whether AL was associated with survival in EC patients. Last, we did not divide the EC patients into two separate sets (one for training set and one for validation set) due to limited sample size.

## CONCLUSIONS

In conclusion, this study addresses a critical gap in the preoperative management of patients with esophageal cancer by developing a novel nomogram model to assess the risk of anastomotic leakage (AL) following esophagectomy. Our findings demonstrate the effectiveness of this diagnostic nomogram, which incorporates key risk factors, in accurately predicting the occurrence of AL in this patient population. By leveraging clinical data and patient characteristics, our nomogram offers clinicians a valuable tool for risk stratification and early intervention, ultimately improving patient outcomes and optimizing postoperative care. Moving forward, further validation and implementation of this nomogram in clinical practice hold the potential to enhance surgical decision-making, personalize treatment strategies, and reduce the burden of AL-associated complications in patients with esophageal cancer. However, due to the limited sample size, patients were not divided into training sets and validation sets, which we will improve in the future.

## Supplementary Materials

Supplementary Figure 1

Supplementary Tables
