# Supplementary material for: A real-world study was conducted to develop a nomogram that predicts the occurrence of anastomotic leakage in patients with esophageal cancer following esophagectomy
Source: Aging (Albany NY). 2024 May 1;16(9):7733–51. doi: 10.18632/aging.205780 (PMC11131977; doi:10.18632/aging.205780)
Supplement: Supplementary Figure 1 [file aging-16-205780-s001.pdf]

SUPPLEMENTARY FIGURE

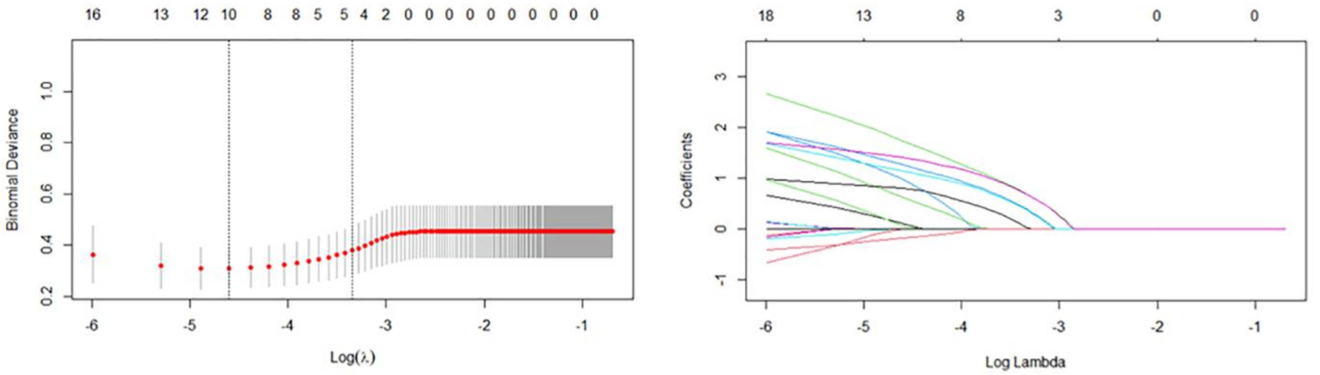

Supplementary Figure 1. LASSO logistic regression analysis to identify candidate predictors for anastomotic leakage.
