# Supplementary material for: A real-world study was conducted to develop a nomogram that predicts the occurrence of anastomotic leakage in patients with esophageal cancer following esophagectomy
Source: Aging (Albany NY). 2024 May 1;16(9):7733–51. doi: 10.18632/aging.205780 (PMC11131977; doi:10.18632/aging.205780)
Supplement: Supplementary Tables [file aging-16-205780-s002.pdf]

SUPPLEMENTARY TABLES

Supplementary Table 1. The severity grading of AL after esophagectomy.

Grade	Definition	Number
A	AL needs no active therapeutic interventions	3
B	AL needs active therapeutic interventions but no surgery	21
C	AL needs surgery	1

Abbreviation: AL: anastomotic leakage.

Supplementary Table 2. The levels of four markers among three groups of AL according to the severity of AL.

Markers	Grade A	Grade B	Grade C*
CRP (mg/l)	228.75 ± 116.16	178.23 ± 51.48	223.68
PCT (ng/ml)	2.07 ± 0.95	5.19 ± 3.04	5.77
IL-6 (pg/ml)	125.67 ± 52.41	209.73 ± 53.51	293.76
IL-10 (pg/ml)	22.23 ± 14.86	13.99 ± 6.43	16.25

Abbreviations: AL: anastomotic leakage; IL-6: interleukin-6; IL-10: interleukin-10; CRP: C-reactive protein; PCT: Procalcitonin.
*We did not conduct statistical analysis, because only one patient was Grade C.
